# Two-step process for disassembly mechanism of proteasome α7 homo-tetradecamer by α6 revealed by high-speed atomic force microscopy

**DOI:** 10.1038/s41598-017-15708-8

**Published:** 2017-11-13

**Authors:** Toshiya Kozai, Taichiro Sekiguchi, Tadashi Satoh, Hirokazu Yagi, Koichi Kato, Takayuki Uchihashi

**Affiliations:** 1 College of Science and Engineering, Kanazawa University, Kakuma, Kanazawa, Ishikawa, 920-1192 Japan; 2 Faculty and Graduate School of Pharmaceutical Sciences, Nagoya City University, 3-1 Tanabe-dori, Mizuho-ku, Nagoya, Aichi 467-8603 Japan; 3 Okazaki Institute for Integrative Bioscience and Institute for Molecular Science, National Institutes of Natural Sciences, 5-1 Higashiyama, Myodaiji, Okazaki, Aichi 444-8787 Japan; 4 Department of Physics, Nagoya University, Furo-cho, Chikusa-ku, Nagoya, Aichi 464-8602 Japan; 5 CREST, JST (Japan Science and Technology), Kawaguchi, Saitama, 332-0012 Japan

## Abstract

The 20S proteasome is a core particle of the eukaryotic proteasome responsible for proteolysis and is composed of layered α and β hetero-heptameric rings. The α7 subunit, which is one of components of the α ring, is known to self-assemble into a double-ringed homo-tetradecamer composed of two layers of the α7 heptameric ring. The α7 tetradecamer is known to disassemble upon the addition of α6 subunit, producing a 1:7 hetero-octameric α6-α7 complex. However, the detailed disassembly mechanism remains unclear. Here, we applied high-speed atomic force microscopy (HS-AFM) to dissect the disassembly process of the α7 double ring caused by interaction with the α6. HS-AFM movies clearly demonstrated two different modes of interaction in which the α6 monomer initially cracks at the interface between the stacked two α7 single rings and the subsequent intercalation of the α6 monomer in the open pore of the α7 single ring blocks the re-association of the single rings into the double ring. This result provides a mechanistic insight about the disassembly process of non-native homo-oligomers formed by proteasome components which is crucial for the initial process for assembly of 20S proteasome.

## Introduction

The proteasome is the central enzyme responsible for regulated proteolysis mostly with ubiquitin-dependent manner in eukaryotic cells^1,2^. The eukaryotic 26S proteasome is a huge protein complex which can be divided into two primary parts, 20S and 19S. The 20S proteasome is a core particle functioning as the catalytic chamber while the 19S complex caps both or either ends of the 20S catalytic chamber and regulates proteolysis activity^3^. The 20S core particle is a barrel-shaped complex composed of two α and two β hetero-heptameric rings stacked of a layer in order of αββα^4–6^. The assembly of the 20S proteasome is initiated by the formation of a heptamer ring with seven homologous and distinct α subunits. The α subunits assemble into the ring form with the well-ordered manner of α1-α2-α3-α4-α5-α6-α7, which is promoted and stabilized by the help of assembly chaperones^7,8^. Subsequently the α-ring serves as a platform on assembly of the seven distinct β subunits in the order of rings β_1-7_ which is accomplished by the help of additional chaperones^9,10^. However, the detailed mechanism for creating a distinctively ordered assembly of the α and β rings and the consequent formation of the 20S proteasome remains elusive.

Among seven α-type subunits in human proteasome, the α7 subunit has a unique feature with which the α7s self-assemble into a double-ringed tetradecamer consisting of two stacked homo-heptameric rings^11,12^, similar to the α ring seen in archaea. Recent combination analysis of mass spectroscopy (MS) and sedimentation velocity analytical ultracentrifugation (SV-AUC) has revealed that the α7 double rings are disassembled into the single ring upon the addition of α6 monomers, resulting in a 1:7 hetero-octamer α6-α7 complex^13^. This implies that disassembly of non-native oligomeric forms of proteasome subunits existing as an assembly intermediate would be crucial toward the terminal 20S form. The following model has been proposed as for disassembly of the α7 double ring^13^: binding of an α6-monomer onto the outer face of an α7 double-ring tetradecamer allosterically induces conformational change of α7 at the interface between the two α7 single rings, which impairs the stacking interaction between the single rings. Nevertheless, the complex form of the α6 monomer and the α7 heptamer was not experimentally verified and further the disassembly mechanisms including validation of the proposed model remain unclear.

Here, we directly observed interaction between the α7 homo-tetradecamer and the α6 monomer by using high-speed atomic force microscopy (HS-AFM), which allows us to study various dynamic behavior of single proteins at work such as conformational change and binding/dissociation events^14–16^. The HS-AFM movies clearly show that the α6 monomer interacts with the open pore of the α7 heptamer ring corresponding to the stacked face of the double ring rather than the outer face. Further, stacking of the two α7-heptamer rings was quite wobbly, engendering a transient cleft between layered α7 single rings. This transient cleft is susceptible to the binding of the α6 monomer onto the α7 single ring’s open-pore site. These observations provide a straightforward view of the disassembly process of the α7 tetradecamer by which the α6 monomer works as a physical blocker against association of the α7 single rings into the double-ring form.

## Results and Discussion

### HS-AFM observation of the human α7 homo-tetradecamer

**Figure 1:**
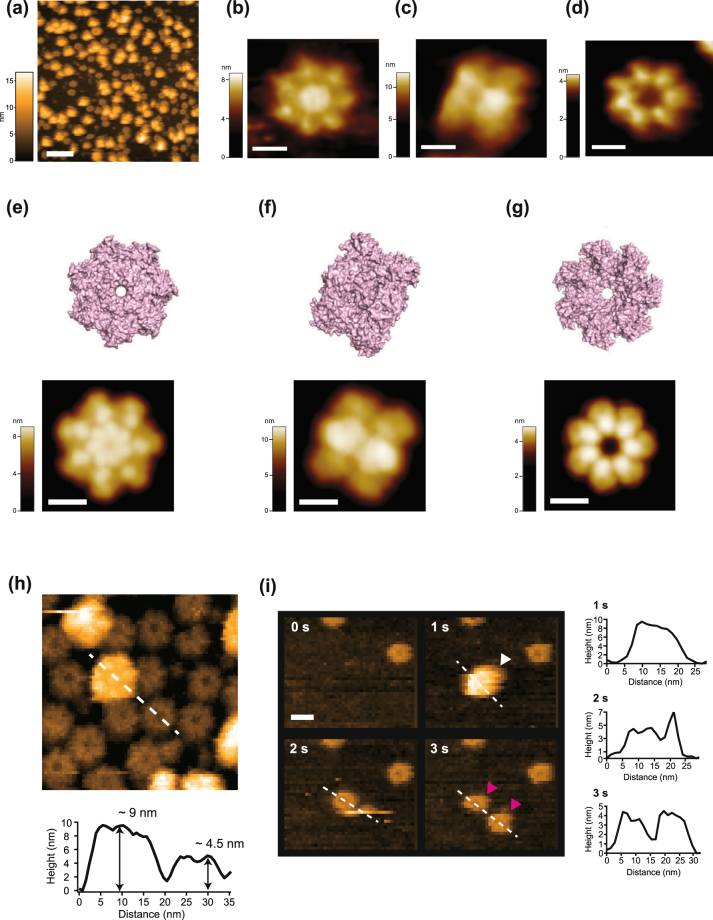
HS-AFM observation of α7 homo-tetradecamer on APTES-mica. (**a**) A wide-area HS-AFM image of α7 homo-tetradecamers on APTES-mica. Scale bar: 50 nm. (**b–d**) Magnified AFM images of three typical orientations of the α7 homo-tetradecamer. Scale bar: 5 nm. (**e–g**) The crystal structures of the α7 homo-tetradecamer (PDB code: 5DSV) at the views of (**e**, upper panel) top and (**f**, upper panel) side. (**g**, upper panel) The crystal structure of α7 homo-heptamer at the view of the open-pore side, which the homo-tetradecamer is bisected at the stacked interface. Simulated AFM images constructed by the crystal structures are shown in the lower panels. Scale bar: 5 nm. (**h**) Height analysis of the α7 double ring and single ring. A height profile on the lower panel corresponds to the cross section indicated by a broken line on the AFM image shown in the upper panel. (**i**) Clipped HS-AFM images capturing the disassembly process of the α7 double ring to the single ring after the adsorption onto the APTES-mica. A white arrow head indicates the double ring, while magenta arrows indicate bisected single rings. Height profiles on the right panels correspond to cross sections indicated by the broken lines on the AFM images at 1 s, 2 s and 3 s. Frame rate, 1 fps. Scale bar: 10 nm.

Figure 1a shows a wide-view HS-AFM image of human-proteasome α7 homo-tetradecamers immobilized on a mica surface chemically treated with 3-aminopropyltriethoxysilane (APTES-mica). The HS-AFM image showed inhomogeneous particles with apparently different height. The diverse structures could be classified into three primary structures including disk (Fig. 1b), barrel-shaped (Fig. 1c) and ring (Fig. 1d) forms. The disk form obviously corresponds to the top surface of the double-ringed tetradecamer displaying the closed gate conformation. In fact, a simulated AFM image constructed by the crystal structure of α7 homo-tetradecamer (PDB code: 5DSV^13^) well reproduced the real HS-AFM image (Fig. 1e). The barrel-shaped form (Fig. 1c) seems to correspond to the side view of the α7 double ring, as is illustrated by a simulated AFM image (Fig. 1f). The third structure (Fig. 1d) showed a clear central pore with a diameter of about 3 nm. This ring-shaped structure appears to be significantly different from the tetradecamer (Fig. 1b). This configuration corresponds to the open-pore side of the α7 heptamer ring, which is turned inwards in the tetradecamer configuration. To illustrate this, a simulated AFM image of the open-pore side is shown in Fig. 1g. Furthermore, the height of the ring was about half of the double ring (Fig. 1h), indicating that the double-ringed tetradecamer was bisected into the single heptamer ring. Apparently, the diameters of the double ring and the bisected single ring are different (Fig. 1h). From the AFM images, the diameter of the double ring is found to be larger than that of the single ring. This is attributed to both the tip-shape convolution and a slight difference of the diameters at the topmost ring faces. The diameter of the taller double ring is affected by the finite and cone-shaped tip more than the single ring (Supplementary Fig. 1).

We assume that the double rings were disassembled into the two single rings during their adsorption onto APTES-mica. To confirm this, we observed adsorption process of the α7 double rings onto APTES-mica by adding the α7 double rings into the observation buffer during HS-AFM imaging (Fig. 1i and Supplementary Movie 1). The double ring was disassembled into two single rings within a single frame (1 s/frame) after the adsorption. This indicates that the strong affinity of the APTES-mica to the closed-pore side of α7 single ring overcomes the stacking interaction between two α7 heptamers. In relative numbers, the orientations of the oligomers were: 11% for the side view, 10% for the top view of the double ring and 79% for the single ring (determined by analyzing 263 oligomers). More than half of the double rings were disassembled by interacting with the substrate.

### Structure of 1:7 hetero-octamer α6-α7 complex

**Figure 2:**
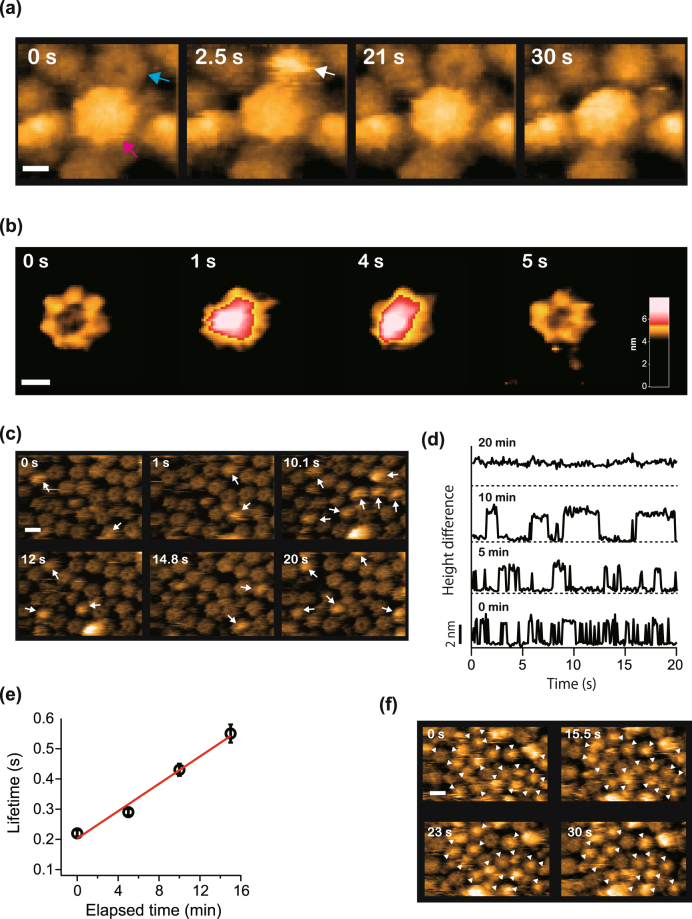
Binding of α6 to α7 homo-heptameric single ring. (**a**) Clipped HS-AFM images displaying binding of the α6 monomer to the open-pore side of the α7 single ring but not to the closed-pore side of the α7 double ring. Cyan and magenta arrows indicate the single and the double rings, respectively. A white arrow indicates binding of the α6 to the open pore of the α7 single ring. Frame rate: 2 fps. Scale bar: 5 nm. (**b**) Closed-up view of the binding of the α6 to the α7 single ring. An area colored red at 1 s and 4 s corresponds to the bound α6. Frame rate: 1 fps. Scale bar: 5 nm. (**c**) Repeated binding and dissociation of the α6 indicated by arrows. The observation was carried out at less than 5 min after the addition of α6 into the observation buffer. Frame rate: 10 fps. Scale bar: 10 nm. (**d**) Time courses of height change of the α7 single ring due to binding and dissociation of the α6 for different elapsed time after the addition of α6. (**e**) Binding lifetime estimated from the dwell time analysis (Supplementary Fig. 1) as a function of elapsed time after the addition of α6. Data plotted are lifetime ± s.d. which was determined by fitting a single exponential function. A red line shows a linear fitting curve. (**f**) Clipped HS-AFM image acquired after 20 min of the addition of α6. Arrow heads indicate the α6 bound to the α7 single ring. Frame rate: 10 fps. Scale bar: 10 nm.

We next examined a binding site of the α6 upon the α7 double ring by directly observing their interaction. Human-proteasome α6 subunits were added into the observation buffer to a final concentration of 1 μM during HS-AFM imaging of the α7 double ring on the APTES-mica. Within a few seconds after the addition of α6, bright spots appeared on the AFM images. Notably, bright spots were never observed on top of the α7 double ring structures, but only on the bisected single rings (Fig. 2a and Supplementary Movie 2). Since it has been addressed that the α6 primarily exits as a monomer form in the solution without forming any oligomer states^13^, the bright spots should correspond to the α6 monomer. Surprisingly, the α6 monomer was repeatedly bound to the open-pore side of the α7 single ring rather than the closed-pore side which is exposed to the solution in the double-ring form.

A magnified image displaying the trapped α6 at the open pore of the α7 single ring is shown in Fig. 2b. The resulting increase of the α7 heptamer’s height was about 2.5 nm. The α7 heptamer harboring the α6 monomer at the pore site should correspond to the 1:7 hetero-octamer α6-α7 complex found by a previous study^13^. The interaction between α6 monomers and α7 heptamers was dynamical with repeated binding and dissociation (Fig. 2c,d and Supplementary Movie 3). Time courses of α7 heptamers’ height increase due to binding of α6 are plotted in Fig. 2d. Interestingly, these plots show that the α6 dwell time is dependent on the time elapsed since the addition of α6. Histograms of the dwell time of α6 on the α7 single ring for an elapsed time of less than 15 min could be fitted well by a single exponential function (Supplementary Fig. 2a). The lifetime of the bound state increases linearly with the total elapsed time (Fig. 2e). When the time after the addition of α6 exceeds 20 min, the lifetime of the bound state becomes significantly longer (Fig. 2f and Supplementary Movie 4). Moreover, the histograms could not be fitted by a single exponential function (Supplementary Fig. 2e and f). The reason for this is that the dwell time for most of the α6 was longer than the maximum imaging frames (1000 frames, 100 s), hampering statistical analysis because of too few binding and dissociation events. Thus, the affinity between the α6 and the α7 single ring gradually changed depending on the time after α6 monomers were added and was nonlinearly boosted after 20 min. The affinity alteration took place only after the addition of α6. Even if α7 single rings were left for several tens of minutes after adsorption to the APTES-mica surface before α6 addition, the time-dependent affinity change between the α6 monomer and the α7 single ring was not affected. This implies that the affinity alteration stems from repeated binding and dissociation of the α6 to the α7 single ring.

There are several reports of proteins that undergo a time-dependent conformational change due to ligand bindings. The interconversion between two different conformational states of such proteins sometimes shows a time scale of several tens of minutes^17–19^. Furthermore, it has been found that fibrinogen gradually changes by immobilization onto the solid substrate, resulting in an enhanced affinity to the substrate^20^. Therefore, we speculate that the origin of the affinity change is caused by time-dependent conformational changes in either the α7 subunits of the heptamer or the α6 monomer, which is induced by their dynamic interaction.

In summary, α6 monomers predominantly interact with the open pore of the α7 single ring but have very little affinity to other sides. Interaction between the α6 monomer and the α7 single ring was dynamical at the onset but the affinity was gradually reinforced, possibly by conformational changes on either the α6 or the α7, which could be elicited by repeated binding and dissociation. Binding of the α6 monomer onto the α7 single ring was eventually strengthened, which would block re-stacking of the α7 single rings.

### Interaction between α6 and the α7 double ring

The fact that α6 interacts with the open-pore side of an α7 single ring implies spontaneous disassembly of the α7 double ring, which raised the question as to how the stacking interaction is fragile. To investigate this issue, we used a bare mica as a substrate with a lower affinity to the α7 double ring than the APTES-mica. The amount of the double rings on the bare mica was increased to 67% from 21% on APTES-mica. However most of them appeared as the barrel shape, indicating the dominant interaction site of the α7 double ring to mica surface was the sidewall of the double ring.

**Figure 3:**
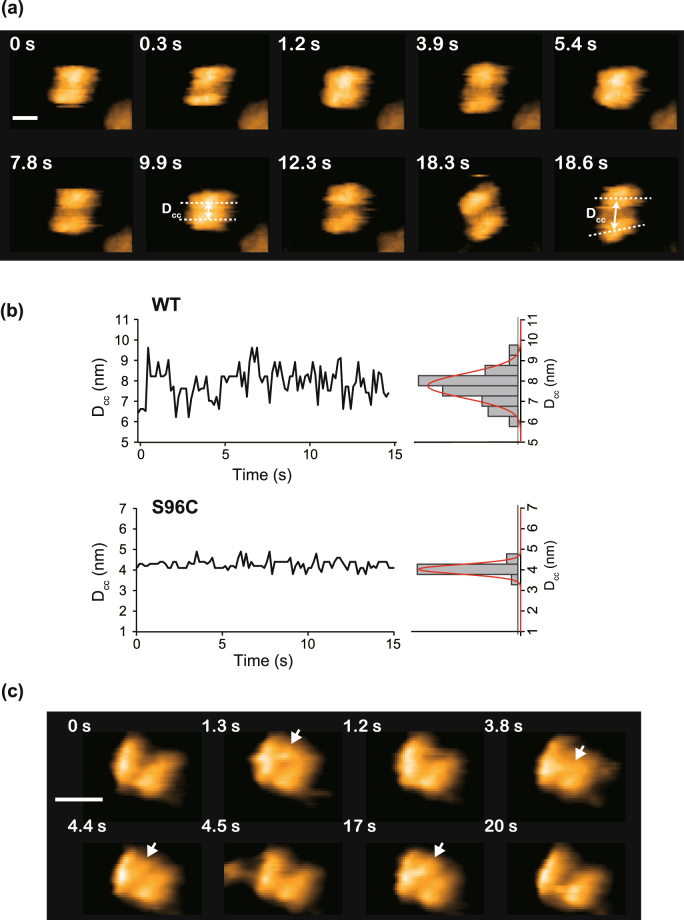
Interaction between α6 and α7 homo-tetradecameric double ring. (**a**) Clipped HS-AFM images demonstrating wobbling of α7 double ring. The interface between the two single rings is loosely packed, resulting in transient cleft. Frame rate: 3.3 fps. Scale bar: 10 nm. (**b**) Time courses of the center-center distance D_cc_ between two single rings for WT and chemically cross-linked S96C double rings. Corresponding histograms are shown on the right (n = 100 for both WT and S96C). Red lines show fitting curves by a Gaussian distribution. The center values ± width for WT and S96C are 8.0 ± 0.9 nm and 4.0 ± 0.3 nm, respectively. A way to estimate of the D_cc_ is indicated by broken lines at 9.9 s and 18.6 s in (**a**). (**c**) Binding and dissociation of the α6 monomer to the cleft between the single rings. Frame rate: 10 fps. Scale bar: 10 nm.

The HS-AFM images revealed an unanticipated wobbling motion of the stacked α7 single rings for ~60% of the α7 double ring forms (Fig. 3a and Supplementary Movie 5), while ~40% of them showed the tightly packed interface as shown in Fig. 1c. A similar wobbling motion was observed for the α7 tetradecamer adsorbed on mica in a different orientation (Supplementary Fig. 3a). The interface between the two rings was perpendicular to the scanning direction of AFM tip. This suggests that the gap formation between the rings is not induced by the scanning of the tip. Center-to-center distance between two single rings (D_cc_) for the wobbling double ring, which corresponds to a distance between two dashed lines drawn on the images at 9.9 s and 18.6 s in Fig. 3a, was fitted by a Gaussian distribution with the center value of about 8 nm and the width of ±0.9 nm (Fig. 3b, upper panel). Furthermore, the α7 double rings sometimes completely dissociated into single rings and then reassembled (Supplementary Movie 6 and Supplementary Fig. 3b). This reassembly of two α7 single rings after the complete dissociation presumably occurs only when the rings are constrained to the surface and, thus, the diffusion is limited to a two-dimensional surface. If the molecules are in a solution, dissociated rings would be shuffled and then associate with a different partner ring. On the other hand, a small-angle neutron scattering (SANS) analysis has revealed no exchange of α7 single rings between double rings in the absence of α6^12,21^. This suggests that the complete dissociation of the double ring is caused by the tip disturbance and the dissociation of the single rings is transient.

When the α6 was added to the observation buffer, the α6 was bound to the cleft at the interface between the two α7 single rings but not to either end of the double ring (Fig. 3c and Supplementary Movie 7). This indicates that α6 favors to interact with the transient cleft at the interface between the two single rings and probably dives into the cleft where the stacking is loose. Here, the question was raised whether α6 can interact with the interface site even if two rings were tightly stacked or not. To investigate this, we prepared chemically cross-linked α7 tetradecamers using the S96C variant and bis(maleimide)ethane. The S96 is placed near the interface and therefore chemical cross-linking through the Cys residues is assumed to stabilize the double ring (Supplementary Fig. 4a). The HS-AFM images of the double ring of the cross-linked S96C variants showed stable double ring forms for all molecules observed even on APTES-mica (Supplementary Fig. 4b). The D_cc_ was about 4 nm which was almost half of that of the WT. Also the width of the distribution with ±0.3 nm was smaller than that of the WT. These values confirm that the double ring was tightly packed with the chemical cross-linking (Fig. 3b, lower panel). In the presence of α6 in the solution, binding of α6 to the interface at the double ring was never observed within the frame time of 0.1 s (Supplementary Movie 8). This suggests that loose interaction with concomitant cleft formation is crucial for the interaction between the α6 monomer and the α7 tetradecamer.

A mass spectroscopy study revealed that the α7 double ring form survived even in the presence of an excess amount of α6, indicating that the homo-tetradecameric and hetero-octameric forms are in equivalent state^13^. This could originate from the populations of the double rings with a tightly or loosely packed interface.

**Figure 4:**
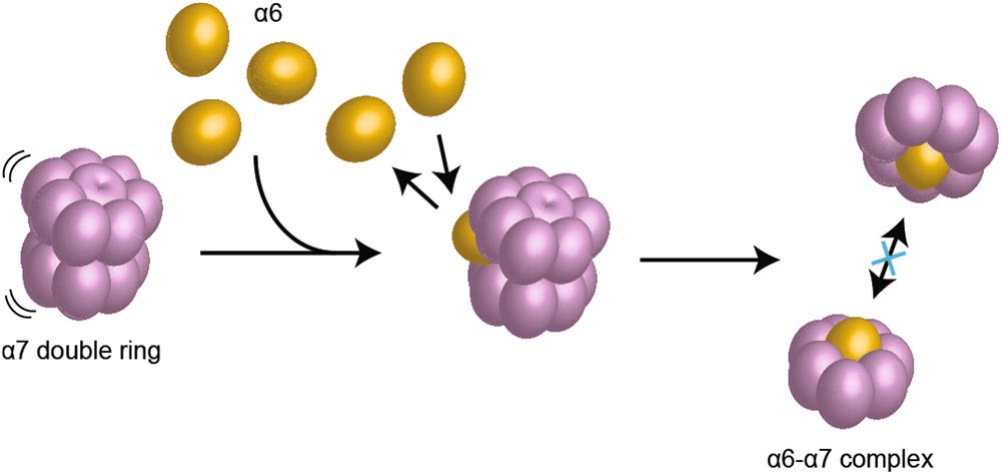
A proposed model of the two-step disassembly process of α7 homo-tetradecamer and following formation of α6-α7 hetero-octamer. As the first step, the α6 monomer encounters the loosely packed interface between the α7 heptamer rings of the tetradecamer ring. Subsequent accommodation of the α6 at the central pore of the heptameric ring due to the enhanced affinity blocks the re-association of the single rings into the double ring, resulting in stable α6-α7 hetero-octamer.

In summary, our HS-AFM results capturing single-molecule dynamics clearly demonstrate the interaction between the self-assembled α7 oligomer and the α6 subunit which are parts of the human 20S proteasome. Based on the HS-AFM imaging, we propose a model for a two-step disassembly mechanism which illustrates a comprehensive view of the disassembly of the α7 homo-tetradecamer induced by the α6 monomer and consequent α6-α7 hetero-octamer (Fig. 4). The stacking interaction of the α7 tetradecamer is inherently unsteady and thus the interface between two α7 single rings creates a transient cleft. When the interface has sufficient clearance, an α6 monomer initially encounters the cleft and breaks open the gap due to a strong affinity to the open-pore side of the α7 single ring, resulting in bisection of the double ring and transient interaction between α6 and the open-pore of the α7 single ring. Then, as the second step, subsequent affinity reinforcement induces tight binding of α6 at the central pore of the single ring. The final formation of the octamer complex impedes the re-assembly of α7 subunits to tetradecamers (Supplementary Movie 9).

Disassembly of homo-oligomers of proteasome α subunits could be essential as the initial stage of the formation of 20S proteasome because distinct seven α subunits may self-assemble into oligomers in a cell. Similar molecular mechanism found in this study would be involved in disassembly homo-oligomer composed of other α subunits. In this context, our study provides the implication of the initial process for assembly of the 20S proteasome. To unveil the full view of the formation process of a proteasome, further comprehensive investigation requires extended to initial disassembly processes of both α and β homo-oligomers and following their assembly to hetero-heptamers with assistance of assembly chaperones. Combination of HS-AFM with other technique such as MS, SV-AUC and SANS should be powerful analytical strategy to this purpose.

## Methods

### Purification of wild-type α7 DR, mutant DR^S96C^ and α6

Human proteasome α6 short isoform and α7 subunits were produced in *Escherichia coli* and purified as previously described^12,13,21^. Briefly, cell lysates were subjected to anion-exchange chromatography (DEAE Sepharose, GE Healthcare) after sonication and centrifugation. The resultant proteins were further purified using anion-exchange and size-exclusion columns with RESOURCE Q and Superdex 200 pg resins (GE Healthcare), respectively.

The S96C mutant was purified the same way as the wild-type α7. For the chemical cross-linking, 500 μl of the purified 0.2 mM S96C-α7 dissolved in 20 mM Tris-HCl (pH 8.0) and 150 mM NaCl was incubated with 10 μl of 10 mM bis(maleimido)ethane (BMOE, Thermo Scientific) at 4 °C for 1 h. To quench and remove the unreacted BMOE, 1 mM DTT was added and subsequently size-exclusion chromatography (Superdex 200 increase, GE Healthcare) was performed.

Purified samples (wild-type α6 and α7 and cross-linked α7 S96C variant) were confirmed to show expected molecular masses by SDS-PAGE (Supplementary Fig. 5).

### High-speed AFM observation

All HS-AFM images shown in the article were taken by a laboratory-built HS-AFM. As an operation mode of AFM, tapping mode was used to minimize disturbance of molecular phenomena by the AFM probe^22,23^. A small cantilever with a length of 7 µm, a width of 2 µm, and a thickness of 90 nm was used. Nominal spring constant, mechanical resonant frequency and quality factor in a solution are ~0.2 N/m, ~800 kHz and ~2, respectively. An amorphous carbon pillar was grown on the original bird-beak tip of the small cantilever by using electron beam deposition (EBD) with the spot mode of a scanning electron microscope^24^. The EBD tip was further sharpened to be less than 4 nm in diameter by plasma etching under argon environment^24^. For HS-AFM imaging, the free oscillation amplitude was set to be ~2 nm and the set-point amplitude for the feedback control to keep the cantilever amplitude constant was ~90% of the free oscillation amplitude, resulting in a few tens of pico-Newton for the tapping force.

As a solid substrate to adsorb α7 tetradecamers, either bare mica or chemically functionalized mica with 3-aminopropyltriethoxy silane (APTES-mica) was used. The APTES-mica was prepared by placing a droplet (3 μl) of 0.01% APTES on a fleshly cleaved mica surface. After 5 min incubation, the APTES solution was thoroughly washed with pure water. A sample droplet (3–5 μl) including α7 tetradecamers was placed on the substrate and incubated for 3 min. Then residual proteins were washed out with the observation buffer (20 mM Tris-HCl (pH 8.0), 150 mM NaCl). After that, the sample stage was immersed into the observation buffer with 70 μl and the HS-AFM imaging was carried out. In some experiments, a solution of α6 monomer was added to the observation buffer so that the final concentration was 1 μM.

### Simulation of AFM Image

Simulated AFM images of an α7 tetradecamer or a heptamer were generated by a simple hard sphere model using the crystal structure (PDB code: 5DSV). For the α7 single ring, one single ring was deleted from the PDB file by using PyMol. An AFM probe was modeled by a cone shape with a radius of 1 nm and half cone angle of 10°. The simulated AFM images were then smoothed by a frequency filter with a cut-off spatial frequency of 3 nm.

### Dwell time Analysis

To estimate binding lifetime (τ) of α6 on the α7 single ring, the bound-state dwell times were measured for successive HS-AFM images. The binding and dissociation events of α6 were determined visually, namely if a bright spot appeared or disappeared between two successive AFM images.

### Analysis of center-center distance between two α7 single rings

On the side–view image of the double ring form, the middle lines at each single ring were drawn as shown in Fig. 3a (images at 9.9 s and 18.6 s). These middle lines were positioned to coincide with the highest points of each ring. The distance between the middle points of the lines was measured.

## Electronic supplementary material

Supplementary Materials

Bisection of the α7 double ring by adsorption on APTES-mica

Interaction between α6 and α7 heptameric ring

Binding and dissociation of α6 to α7 heptamer just after the addition of α6

Binding and dissociation of α6 to α7 heptamer 20 min after the addition of α6

Wobbling motion of the stacked α7 single rings

Transient dissociation of the α7 single rings

Binding and dissociation of α6 to the cleft between α7 single rings

Non-binding of α6 to cross-linked α7-S96C double ring

Binding of α6 to α7 double ring and disassembly of the double ring
