# Supplementary material for: Two-step process for disassembly mechanism of proteasome α7 homo-tetradecamer by α6 revealed by high-speed atomic force microscopy
Source: Sci Rep. 2017 Nov 13;7:15373. doi: 10.1038/s41598-017-15708-8 (PMC5684232; doi:10.1038/s41598-017-15708-8)
Supplement: Supplementary file 1 — Supplementary Materials [file 41598_2017_15708_MOESM1_ESM.pdf]

# Two-step process for disassembly mechanism of proteasome α7 homo-tetradecamer by α6 revealed by high-speed atomic force microscopy

**Toshiya Kozai[1], Taichiro Sekiguchi[2], Tadashi Satoh[2], Hirokazu Yagi[2], Koichi Kato[2, 3, *], and Takayuki Uchihashi[4,5] ***

[1]College of Science and Engineering, Kanazawa University, Kakuma, Kanazawa, Ishikawa 920-1192, Japan

[2]Faculty and Graduate School of Pharmaceutical Sciences, Nagoya City University, 3-1 Tanabe-dori, Mizuho-ku, Nagoya, Aichi 467-8603, Japan

[3]Okazaki Institute for Integrative Bioscience and Institute for Molecular Science, National Institutes of Natural Sciences, 5-1 Higashiyama, Myodaiji, Okazaki, Aichi 444-8787, Japan

[4]Department of Physics, Nagoya University, Furo-cho, Chikusa-ku, Nagoya, Aichi 464-8602, Japan

[5]CREST, JST (Japan Science and Technology), Kawaguchi, Saitama 332-0012, Japan

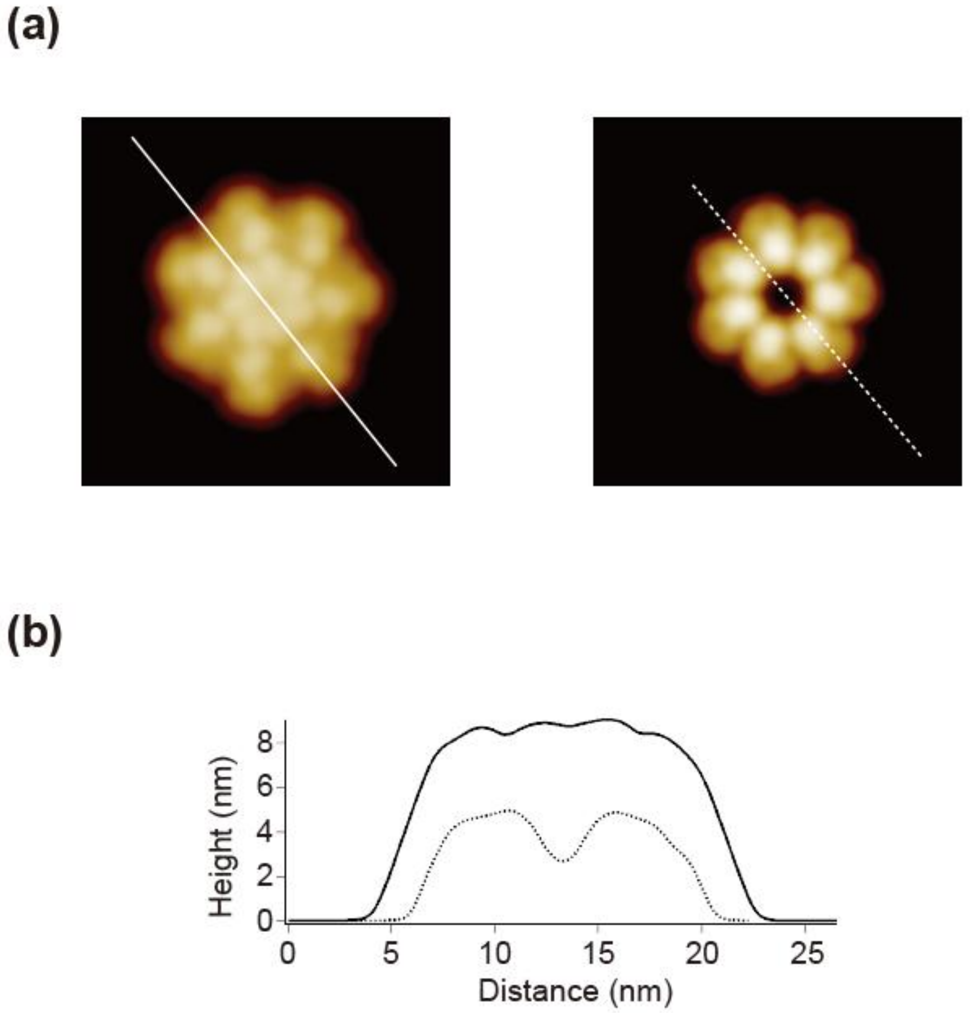


**Figure S1. Comparison between simulated images of α7 tetradecamer and heptamer.** (a) Simulated images of the α7 (left) tetradecamer and (right) heptamer. (b) Corresponding height profiles indicated by the lines in (a). The solid and broken lines correspond to the profiles for the double ring and the single ring, respectively.

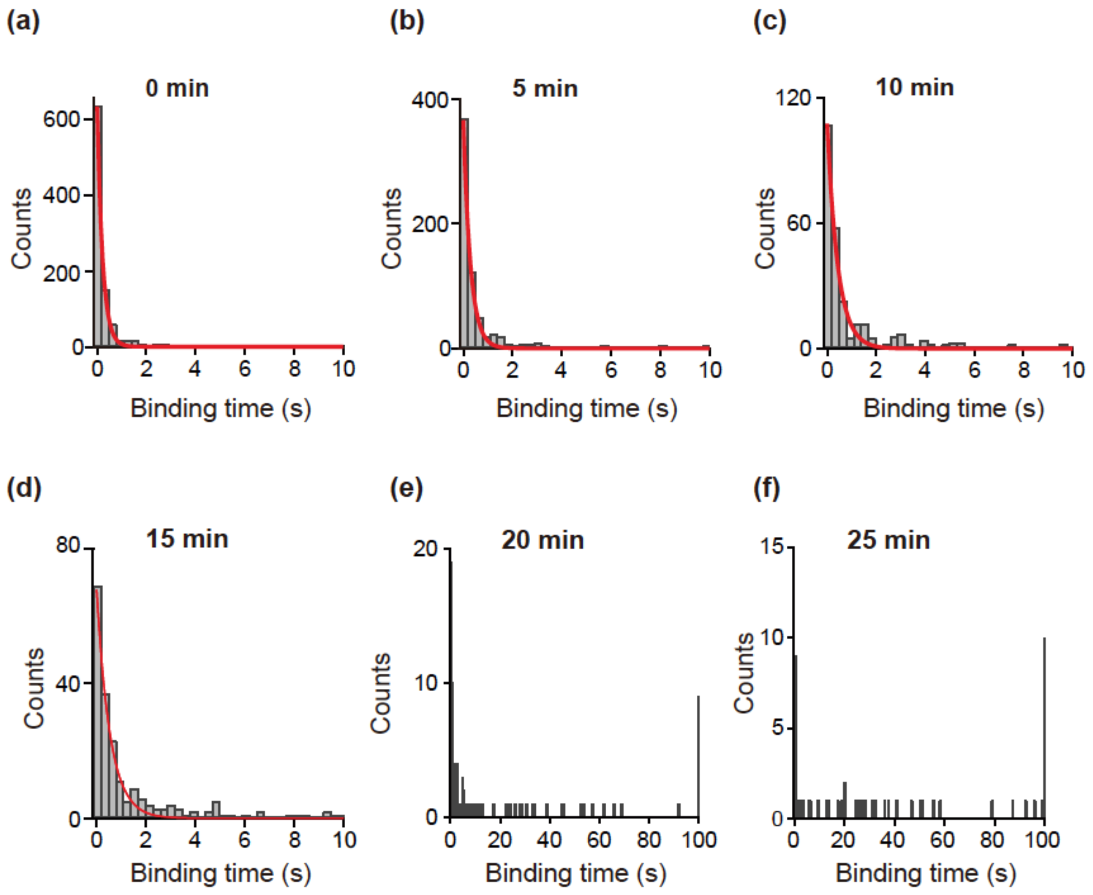


**Figure S2. Dwell time analysis of α6 on α7 homo-heptamer.** Histograms of dwell time of α6 bound to the α7 heptamer ring for a delimited elapsed time after the addition of α6. The delimited elapsed time, $t_e$, was (**a**) $0 \le t_e < 5$ min, (**b**) 5 min $\le t_e <$ 10 min, (**c**) 10 min $\le t_e <$ 15 min, (**d**) 15 min $\le t_e <$ 20 min, (**e**) 20 min $\le t_e <$ 25 min and (**f**) 25 min $\le t_e$. Red lines in (**a-d**) represent fitted curves with single exponential functions, giving the life time ± s.d.; (a) $\tau_{0\ min} = 0.22 \pm 0.01$ s (n = 970), (b) $\tau_{5\ min} = 0.29 \pm 0.01$ s (n = 712), (c) $\tau_{10\ min} = 0.43 \pm 0.02$ s (n = 283), (d) $\tau_{15\ min} = 0.55 \pm 0.03$ s (n = 235). (**e** and **f**) Over 20 min, the dwell time was significantly longer and histograms were not able to be fitted by single exponential curves. Mean binding times ± s.ds. were (**e**) 19 ± 31s at 20 min $\le t_e <$ 25 min (n = 100) and (**f**) 33 ± 38s at 25 min $\le t_e$ (n = 68).

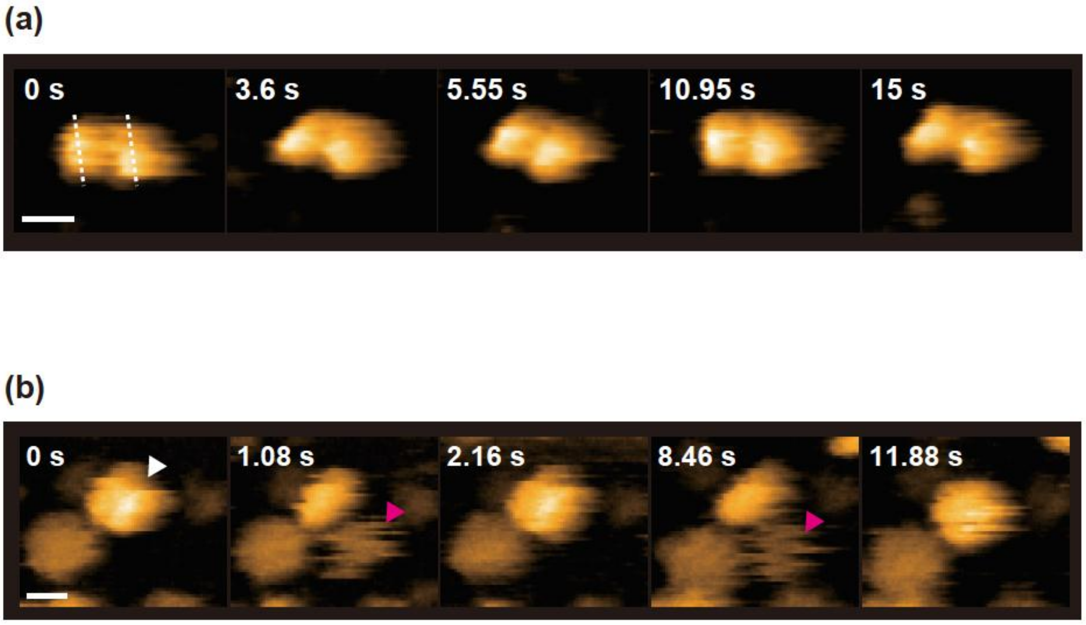


**Figure S3. Weak stacking interaction between the stacked α7 single rings.** (a) The double ring is adsorbed on the substrate with an orientation where the ring face is perpendicular to the scanning direction. Wobbling motion of the α7 rings is still observed. Scale bar, 10 nm. (b). Complete dissociation of the α7 rings and reassembly to the double ring. A white arrow head indicates the double ring, while magenta arrow heads indicate the dissociated single ring. Scale bar, 10 nm.

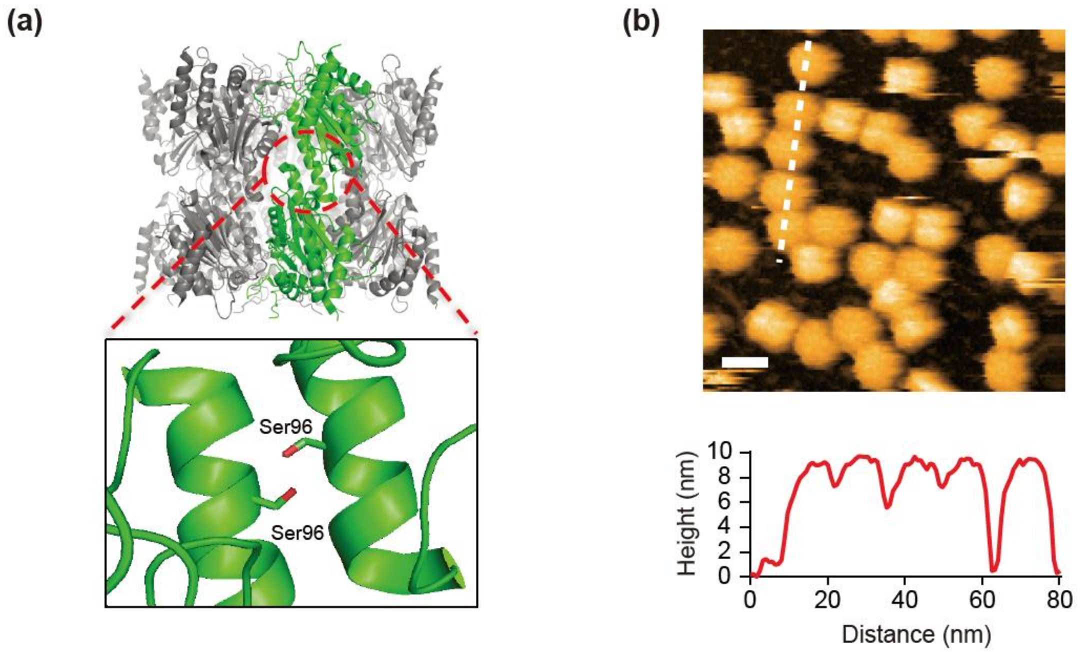


**Figure S4. Schematic of position of Ser96 in α7 and AFM image of cross-linked S96C double rings. (a)** Structure of the wild-type α7 double ring showing positions of S96 residues, which were replaced by Cys residues. Chemically cross-linked α7 double ring was prepared using the S96C variant. **(b)** HS-AFM image of cross-linked S96C double rings, which are stable even on the APTES-mica.

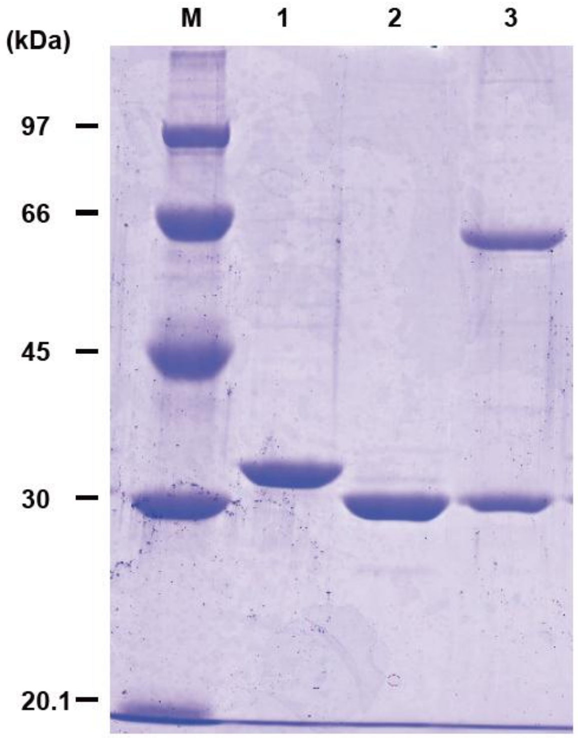


**Figure S5. Purities of proteasome α6 and α7 subunits.** Purified samples were analyzed by SDS-PAGE; M: molecular weight of marker proteins, lane 1: α6 subunit, lane 2: α7 subunit, lane 3: chemically cross-linked α7 S96C variant.

**Supplementary Movies**

**Movie 1. Bisection of the α7 double ring by adsorption on APTES-mica.** Acquired frame rate: 1 fps. The movie is x 1 playback.

**Movie 2. Interaction between α6 and α7 heptameric ring.** Acquired frame rate: 2 fps. The movie is x 3 playback.

**Movie 3. Binding and dissociation of α6 to α7 heptamer just after the addition of α6.** Acquired frame rate: 10 fps. The movie is x 1 playback.

**Movie 4. Binding and dissociation of α6 to α7 heptamer after 20 min of the addition of α6.** Acquired frame rate: 10 fps. The movie is x 2 playback.

**Movie 5. Wobbling motion of the stacked α7 single rings.** Acquired frame rate: 3.3 fps. The movie is x 2 playback.

**Movie 6. Transient dissociation of the α7 single rings.** Acquired frame rate: 6.6 fps. The movie is x 1 playback.

**Movie 7. Binding and dissociation of α6 to the cleft between α7 single rings.** Acquired frame rate: 10 fps. The movie is x 1 playback.

**Movie 8. Non-binding of α6 to cross-linked α7-S96C double ring.** Acquired frame rate: 10 fps. The movie is x 1 playback.

**Movie 9. Binding of α6 to α7 double ring and disassembly of the double ring.** Acquired frame rate: 6.6 fps. The movie is x 2 playback.
